# A Novel Technique for the Preparation of Iron Carbide and Carbon Concentrate from Blast Furnace Dust

**DOI:** 10.3390/ma15228241

**Published:** 2022-11-20

**Authors:** Dong Chen, Hongwei Guo, Peng Li, Feibao Wu, Yanan Lv, Bingji Yan, Wei Zhao, Yifan Su

**Affiliations:** 1 School of Iron and Steel, Soochow University, Suzhou 215131, China; 2 Department of Mechanical and Electrical Engineering, Suzhou Institute of Industrial Technology, Suzhou 215104, China

**Keywords:** blast furnace dust, iron carbide, carbon concentrate, carburization, magnetic separation, acid leaching

## Abstract

Blast furnace (BF) dust is a typical refractory iron resource. A novel technology-based utilization of BF dust as iron carbide and carbon concentrate by applying carburization roasting followed by magnetic separation and acid leaching is proposed. Under optimized conditions, an electric arc furnace (EAF) burden assaying 80.79% Fe and 7.63% C with a corresponding iron recovery rate of 87.26% and a carbon concentrate assaying 67.06% C with a corresponding carbon recovery rate of 81.23% were prepared. Furthermore, the carburization behavior and separation mechanism were revealed using X-ray powder diffraction, scanning electron microscopy, and optical microscopy. The results show that the separation efficiency of iron carbide, gangue, and carbon is very low. Na_2_SO_4_ is a highly effective additive to strengthen the separation efficiency as it can enhance the carburization index, enlarge the iron carbide particle size, improve the embed embedded relationship of iron carbide and gangue, and promote the gangue leaching efficiency. The study demonstrates that preparation of iron carbide and carbon concentrate from BF dust using the proposed technology is a feasible method.

## 1. Introduction

With fast development of the economy, the iron and steel industry is expanding quickly and the production of steel is increasing remarkably. The world crude steel output increased to 1.95 billion tons and nearly 1.03 billion tons of steel was produced in China in 2021. Owing to the massive production of steel, large amounts of dust are produced during the process of iron and steel production, especially in the ironmaking process. These dusts contain not only abundant Fe, but also considerable Zn, Pb, K, Na, and so on [1,2].

Blast furnace (BF) dust is a significantly secondary resource that is discharged from the top of BF with BF gas. Each ton of iron is supposed to produce approximately 20 kg BF dust, and about twenty million tons of BF dust in the iron works is produced in China annually [3]. In general, BF dust contains not only abundant Fe, but also C, Zn, Pb, As, Cd, Ti, Mn, and so on [4,5]. Owing to the high content of these valuable elements, it is important to utilize BF dust.

Currently, BF dust is usually used as a raw material to partially replace iron ore to produce sinter by sintering for BF ironmaking [5]. Owing to the excessive use of BF dust, the zinc and lead contents of BF burdens significantly increase and the iron content of BF burden decreases [6], which increases the BF energy consumption, decreases the BF utilization coefficient, corrodes the BF lining, and impedes the BF smooth running [7]. To utilize BF dust, beneficiation technologies such as magnetic separation, flotation separation, and gravity separation have been adopted to recove Fe and C [8,9,10]. However, BF dust has poor floatability, a fine dust grain size, low density, and a complex mineral structure and composition, so it is difficult to separate Fe, C, Zn, and Pb from gangue with the aforementioned methods. In order to improve the separation efficiency, Ju et al. developed a technique called magnetization roasting–magnetic separation [11]. With this treatment, the separation efficiency of iron and gangue dramatically increased; however, Zn and Pb also were difficult to separate from BF dust, which was still harmful to BF smelting. 

To efficiently utilize BF dust, direct-reduction processes, such as the rotary kiln process [12] and rotary hearth furnace process [13], have been developed. Although Fe, C, Zn, and Pb in BF dust can be recycled using these treatments, the iron metallization degree and iron grade of product are low. In order to improve the product quality, the smelting reduction process [14] and direct reduction–magnetic separation process [15] have been adopted to produce molten iron and reduced iron, respectively. However, owing to the low iron grade of BF dust, energy consumption of the process using smelting reduction is high. Huang et al. [16] reported that reduced iron, obtained from direct reduction process, was easily oxidized during the grinding process, which would decrease the quality of the product using the direct reduction–magnetic separation process. Compared with reduced iron, iron carbide is difficult to oxidize. Furthermore, iron carbide possesses a low content of iron oxide, fast forming foaming slag in smelting, and the potential to serve as an auxiliary heat [17]. Therefore, iron carbide is a better burden for EAF than reduced iron. In addition, owing to the low carburization temperature (600–800 °C), Zn and Pb may remain in the iron carbide. During the EAF smelting, Zn and Pb can be enriched in EAF dust, which are easy to recycle [18]. Thus, if BF dust can be prepared as iron carbide, the utilization of BF dust may be further improved.

Owing to abundant gangue in BF dust, the iron carbide prepared from BF dust cannot be used for EAF steelmaking. Ye et al. [19] found that saturation magnetization of iron carbide is up to 150 emu/g. According to previous findings [20], magnetic separation is a highly effective method to separate magnetic mineral from gangue. However, both carbon and gangue enter the magnetic separation tailing after magnetic separation, which leads to the low carbon content of carbon concentrate. The low-grade carbon concentrate is difficult to utilize. Based on the findings of the previous study [21], sodium salts can dramatically increase the liberation degree of iron minerals and gangue, which greatly promotes the separation of iron minerals and gangue. The grade of iron minerals and iron recovery rate remarkably increase during the magnetic separation. In addition, the research found [22] that sodium salts could significantly enhance the dissolution of gangue in acid during the acid leaching. Therefore, a new technique based on carburization followed by magnetic separation and acid leaching was developed to produce iron carbide and carbon concentrate from BF dust in this study. To further improve the separation efficiency, sodium salts were used and their mechanism of action was also investigated. 

## 2. Materials and Methods

### 2.1. Materials

**Figure 1:**
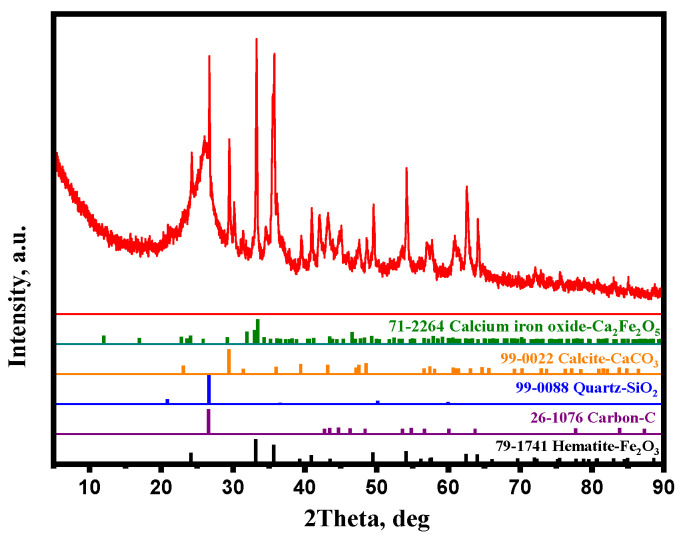
XRD pattern of BF dust.

In this study, a gas mixture consisting of CO, CO_2_, H_2_, and N_2_ with a purity of >99.9% was used. BF dust and Na_2_SO_4_ analytical reagent were used as raw materials. The BF dust was collected from Shandong Iron and Steel Group. Table 1 shows the main chemical composition of BF dust, in which the contents of Fe, C, SiO_2_, Al_2_O_3_, and CaO are 32.25%, 34%, 6.03%, 2.95%, and 4.03%, respectively. According to the results of Table 1 and Figure 1, there is a large amount of carbon, abundant carbonas (CaCO_3_), and a small number of sulfides. In addition, a small amount of limonite and magnesium carbonate may exist in the BF dust. As the result, LOI is up to 37.25%. The BF dust is fine, with 100% passing through 0.075 mm. The X-ray diffraction (XRD) pattern of BF dust (Figure 1) shows that the main iron-bearing mineral is Fe_2_O_3_ and the main gangues are calcite and quartz.

**Figure 2:**
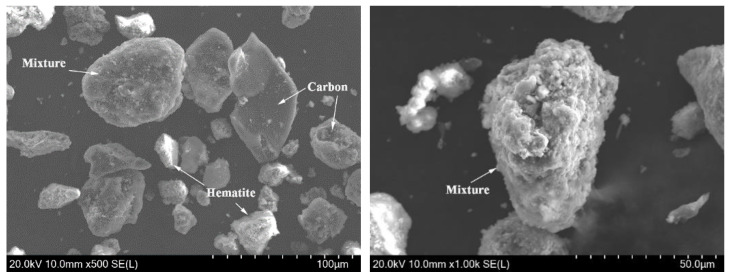
Morphology of BF particles under SEM (carbon, hematite, and mixture particles were detected by EDS).

The morphology of a BF dust particle under scanning electron microscope (SEM) is shown in Figure 2. The hematite, carbon, and mixture particles were identified by energy-dispersive spectrometry (EDS). It is clear that some hematite and carbon particles are individual particles, which may be easy to separate after carburization. However, some particles are mixture particles, which is mainly composed of carbon, hematite, calcium iron oxide, quartz, calcite, and so on. These mixture particles are very likely to be formed under a high-temperature process inside a blast furnace. Owing to the high-temperature function, the iron minerals, carbon, and gangue combine closely inside mixture particles, which leads to difficult in the separation and purification of iron minerals and carbon.

### 2.2. Experimental

#### 2.2.1. Carburization Roasting/Magnetic Separation/Acid Leaching Process

Details of the pelletization, carburization, milling, and magnetic separation processes can be found in the literature [23]. Green balls (Φ 8–10 mm) were made using a mixture of BF dust and additives in a disc pelletizer. The dried pellets were preheated in a preheating tank (Φ 40 × 600 mm) by roasting at 1000 °C for 15 min under N_2_ gas with a flow rate of 1.5 L/min, after which the preheated pellets were cooled under N_2_ gas with a flow rate of 1 L/min. The carburizing tank (Φ 40 × 600 mm) containing the preheated pellets (50 g) was placed in a shaft furnace (Φ 80 × 1000 mm) and carburized in a mixture of 75% CO-25% H_2_ at a flow rate of 1.2 L/min. The carburized pellets were then cooled under N_2_ gas at a flow rate of 1 L/min. The carburized pellets were ground to a particle size of 90.30%, less than 10 μm, in a conical mill (Φ 160 × 60 mm) with a pulp density of 50%. After that, the pulp was magnetically separated in a magnetic tube at a magnetic field strength of 130 mT. The magnetic separation tailing was leached in a 250 mL conical flack equipped with a mechanical stirrer at a stirring rate of 500 rpm, and the leaching conditions were fixed at a liquid-to-solid ratio of 10:1 and leaching time of 60 min. Lately, the acid-leached concentrates were filtered, washed, and dried.

#### 2.2.2. Characterization

Chemical analysis of iron and ferrous was carried out using ISO 2597-1-2006 standard and ISO 9035-1989 standard, respectively. An inductively coupled plasma emission spectrometer (ICP, 5100, Agilent, Santa Clara County, CA, USA) was used to measure the chemical composition, and a carbon-sulfur infrared analyzer (CSI, HCS-801, Science, Deyang, China) was adopted to detect the carbon content. The free carbon content was determined as described in the literature [24]. The combined carbon content was calculated by subtracting the total carbon content from the free carbon content. The phases of the samples were analyzed using XRD (Ultima IV, Rigaku, Tokyo, Japan) with Cu K_α_ (λ = 1.5405 Å) radiation at a step size of 0.02° and a scan rate of 10°/min. The microstructure and morphology of the samples were detected by SEM (SU5000, Hitachi, Tokyo, Japan) and EDS (X-MAX 20, Oxford, UK).

The carburizing efficiency was expressed by the carburization index and the carburization index was calculated by Equation (1):

(1) $$\eta =\frac{m_{c}/12.01}{m_{\mathrm{Fe}}/55.85}\times 100$$

where *η* is the carburization index; *m_c_* is the combined carbon content in carburized pellets (%); *m_Fe_* is the total iron content in carburized pellets (%); and 12.01 and 55.85 are the molar mass of carbon and iron (g/mol), respectively.

To investigate the iron recovery efficiency, the iron recovery rate was calculated using Equation (2):

(2) $$\varepsilon =\frac{m_{m}}{m_{p}}\times \frac{n_{Fe}}{m_{Fe}}$$

where *ε* is the iron recovery rate (%), *m_p_* is the mass of the carburized pellets (g), *m_m_* the mass of the magnetic concentrate (g), and *n_Fe_* is the total iron content in magnetic concentrate (%).

To investigate the carbon recovery efficiency, the carbon recovery rate was calculated using Equation (3):

(3) $$\gamma =\frac{m_{a}}{m_{p}}\times \frac{n_{c}}{N_{c}}$$

where *γ* is the carbon recovery rate (%), *m_a_* is the mass of the acid leaching concentrate (g), *m_p_* is the mass of the carburized pellets (g), *n_c_* is the free carbon content in acid leaching concentrate (%), and *N_c_* is the free carbon content in carburized pellets (%).

## 3. Results and Discussion

### 3.1. Preparation of Iron Carbide

#### 3.1.1. Effect of Na_2_SO_4_

**Figure 3:**
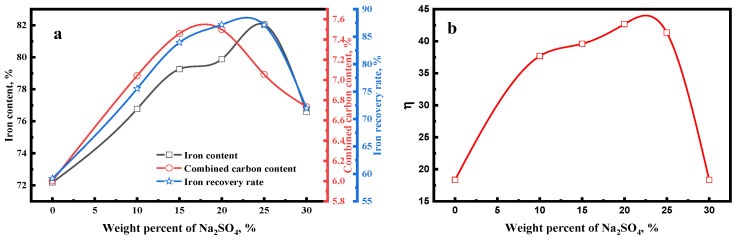
Effect of Na_2_SO_4_ on the (**a**) magnetic concentrate and (**b**) carburization index of carburized pellet (carburized at 650 °C for 150 min).

Figure 3a shows the effect of Na_2_SO_4_ on the magnetic concentrate. The iron content and iron recovery rate of magnetic concentrate particularly increased when the weight percent of Na_2_SO_4_ went up to 25%, after which all these parameters decreased when the weight percent of Na_2_SO_4_ exceeded 25%. The combined carbon content of magnetic concentrate dramatically increased with the increasing weight percent of Na_2_SO_4_, but decreased when the weight percent of Na_2_SO_4_ exceeded 20%.

**Figure 4:**
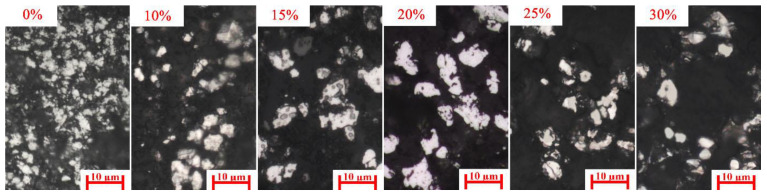
Microstructures of the carburized pellet as a function of Na_2_SO_4_ (iron carbide-white).

Owing to the low carburization temperature, the carburization efficiency of iron minerals is low [24]. Moreover, Figure 2 shows that part of iron mineral particles was wrapped in the mixture particles, which also hindered the carburization of iron mineral particles. Thus, the carburization index was only 18.36 without Na_2_SO_4_ additive, which indicated the low carburization efficiency. From Figure 4, the size of the iron carbide particles without Na_2_SO_4_ was very fine. Our previous study [23] reported that fine iron carbide was difficult to separate from gangue. In addition, owing to the existence of mixture particles, the iron mineral particles and gangue particles were more difficult to dissociate. Owing to the low carburization efficiency, fine iron carbide particles, and close combination of iron minerals and gangue, the separation of iron carbide and gangue was difficult. Therefore, the iron content, combined carbon content, and iron recovery rate of the magnetic concentrate without Na_2_SO_4_ additive were only 72.18%, 6.0%, and 59.17%, respectively.

When Na_2_SO_4_ was added in the pellet, the carburization index dramatically increased (Figure 3b). Moreover, the carburization index increased from 18.36 to 42.67 when the weight percent of Na_2_SO_4_ increased from 0 to 20%. According to the study by Iguchi et al. [25,26], the reactions for the carburization in CO-H_2_ mixture gas are represented as follows:

(4) CO→O(ad)+[C]

(5) $$O\left(ad\right)+CO=CO_{2}$$

(6) $$O\left(ad\right)+H_{2}=H_{2}O$$

(7) $$3Fe\left(s\right)+\left[C\right]=Fe_{3}C\left(s\right)$$

(8) $$5Fe_{3}C\left(s\right)+\left[C\right]=3Fe_{5}C_{2}\left(s\right)$$

The control step of the carburization reaction is the dissociative adsorption of CO molecules (Equation (4)) on the iron surface in the temperature range from 500 to 750 °C. Ribeiro and co-workers [27] found that alkali metal ions (Na^+^ ions) accelerated the dissociative adsorption of CO molecules on the surface of iron. In this study, Na_2_SO_4_ enhanced the dissociative adsorption of CO molecules during the carburizing process, thereby improving the carburization reactions of BF dust pellets.

**Figure 5:**
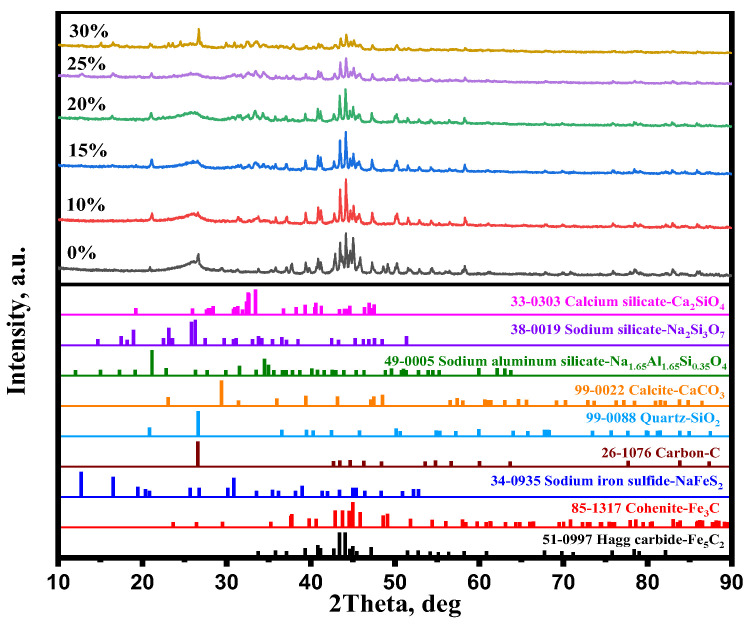
XRD patterns of carburized pellets with various Na_2_SO_4_ weight percentages.

The research [28] found that Na^+^ ion doping can induce the lattice distortion of iron minerals. Lattice distortion improves the diffusion of iron and carbon atoms, as demonstrated by Abdrakhimov et al. [29]. The rapid diffusion of iron and carbon atoms promoted carburization reactions (Equations (7) and (8)). As shown in Figure 5, the main iron mineral diffraction peaks were Fe_3_C and Fe_5_C_2_ without Na_2_SO_4_ additive, whereas the diffraction peak intensity of Fe_3_C significantly decreased and the diffraction peak intensity of Fe_5_C_2_ dramatically increased when Na_2_SO_4_ was added. It is clearly found that Na_2_SO_4_ significantly improved Equation (8). Therefore, the carburization index significantly increased when adding Na_2_SO_4_.

Owing to the rapid diffusion of iron and carbon atoms, the recrystallization of iron and iron carbide grains was promoted, which enhanced the growth of iron carbide particles during the carburization process. Consequently, the size of iron carbide particles increased significantly (Figure 4). In addition, the size of iron carbide particles increased with the increase in the weight percent of Na_2_SO_4_.

**Figure 6:**
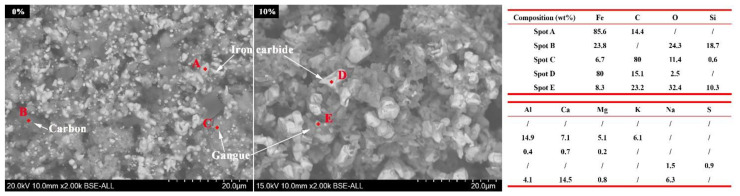
SEM-EDS images of the pellets carburized at 650 °C for 150 min with different weight percentages of Na_2_SO_4_ (iron carbide: white; gangue: gray; carbon and pore: black).

Figure 5 shows that the main gangue components were quartz and calcite in the carburized pellet without Na_2_SO_4_. The previous study [21] reported that the Na_2_SO_4_ could easily react with SiO_2_ and Al_2_O_3_ (sodium modification reactions) during the high-temperature roasting process, thus new gangues such as Na_2_Si_3_O_7_, Na_1.65_Al_1.65_Si_0.35_O_4_, and Ca_2_SiO_4_ were generated (Figure 5) when the Na_2_SO_4_ additive was added. Because of the formation of new gangues, the embedded relationship of iron carbide and gangue particles could be improved (Figure 6), thereby enhancing the separation efficiency of iron carbide and gangue. High carburization efficiency, large iron carbide particle, and a good embedded relationship of iron carbide and gangue dramatically improved the separation efficiency of iron carbide and gangue. As a result, the iron content, combined carbon content, and iron recovery rate of the magnetic concentrate dramality increased from 72.18%, 6.0%, and 59.17%, respectively, to 79.88%, 7.50%, and 87.18%, respectively, when the weight percent of Na_2_SO_4_ increased from 0% to 20%.

When the weight percent of Na_2_SO_4_ reached 30%, the iron content, combined carbon content, and iron recovery rate of the magnetic concentrate decreased to 76.57%, 6.73%, and 71.95%, respectively. During the preheating process (1000 °C), solid Na_2_SO_4_ transformed into liquid Na_2_SO_4_. Owing to too much liquid Na_2_SO_4_, part of the iron surface was coated with abundant Na_2_SO_4_, which may in turn hinder the dissociative adsorption of CO on iron surface during the carburization. Thus, the carburization index significantly decreased (Figure 3b) when the weight percent of Na_2_SO_4_ was >20% and, consequently, the combined carbon of magnetic concentrate decreased. Figure 5 shows that the diffraction peak of NaFeS_2_ appeared when the weight percent of Na_2_SO_4_ reached 20%, and its intensity increased with the increase in the weight percent of Na_2_SO_4_. It is evident that part of the iron mineral transformed into NaFeS_2_, which hindered the carburization of the pellets. Thus, the iron content and iron recovery rate decreased. 

#### 3.1.2. Effect of Carburization Temperature

**Figure 7:**
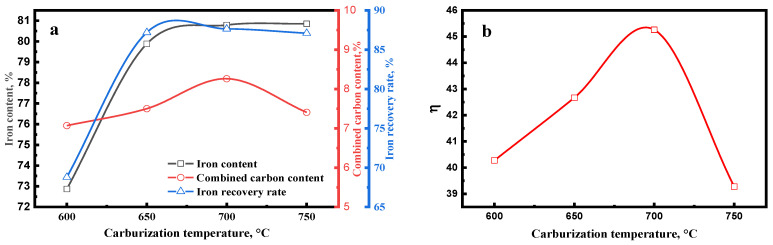
Effect of carburization temperature on the (**a**) magnetic concentrate and (**b**) carburization index of carburized pellet (carburized for 150 min with 20% Na_2_SO_4_).

**Figure 8:**
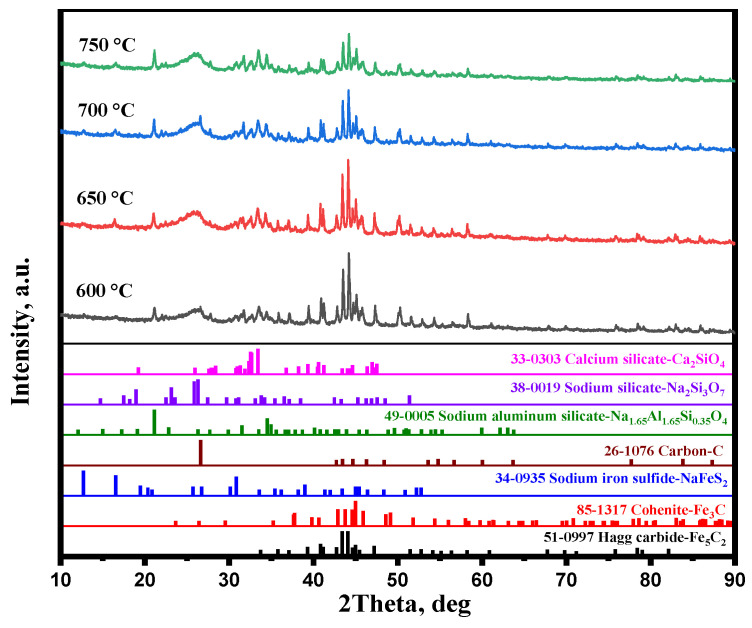
XRD patterns of carburized pellets with different carburization temperatures.

Figure 7a shows the effect of the carburization temperature on the magnetic concentrate. The iron content, combined carbon content, and iron recovery rate of magnetic concentrate increased with the rise in carburization temperature, whereas the combined carbon and iron recovery rate slightly decreased when the carburization temperature was over 700 °C. Iguchi et al. [25] have verified that the carburization of iron ore is controlled by chemical reaction at the temperature of 500 to 750 °C. Therefore, a high temperature significantly improved the carburization rate, thus the carburization index significantly increased at the temperature from 600 to 700 °C (Figure 7b). From Figure 8, it is observed that the diffraction peak intensity of iron carbide increased with the increasing carburization temperature. A high iron carbide content improved the separation of iron carbide and gangue.

**Figure 9:**
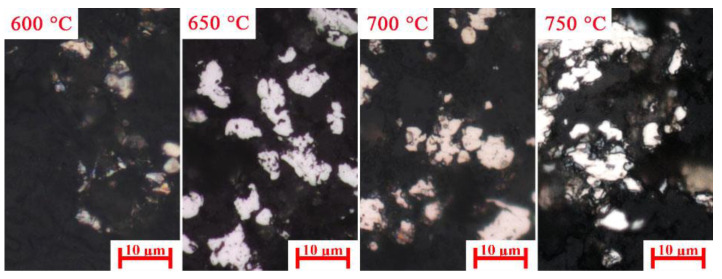
Microstructures of carburized pellets at different carburization temperatures (iron carbide: white).

A previous study [21] proved that a high temperature improved the sodium modification reactions. As shown in Figure 8, the diffraction peak intensity of Na_2_Si_3_O_7_, Na_1.65_Al_1.65_Si_0.35_O_4_, and Ca_2_SiO_4_ increased with the increase in carburization temperature. These high gangues contents enhanced the embedded relationship of iron carbide and gangue. Figure 9 shows that the iron carbide particle size increased with the increasing carburization temperature, which can improve the separation of iron carbide and gangue. Therefore, the high iron carbide content, large iron carbide particle, and good embedded relationship of iron carbide and gangue significantly enhanced the separation of iron carbide and gangue. When the carburization temperature increased from 600 °C to 700 °C, the iron content, combined carbon content, and iron recovery rate of magnetic concentrate increased from 72.87%, 7.07%, and 68.79%, respectively, to 80.79%, 8.26%, and 87.64%, respectively.

When the carburization temperature increased to 750 °C, the sodium modification reactions and iron carbide particle size continued to be improved (Figure 8 and Figure 9). However, the thermodynamic calculation [23] shows that a high temperature hinders the carburization reaction, thus the carburization index decreased when the carburization temperature was over 700 °C (Figure 7b). Therefore, the large iron carbide particle and efficient sodium modification reactions improved the separation of iron carbide and gangue and, consequently, the iron content slightly increased when the carburization temperature reached 750 °C. Owing to the low carburization index, the combined carbon content and iron recovery rate of magnetic concentrate decreased.

#### 3.1.3. Effect of Carburization Time

**Figure 10:**
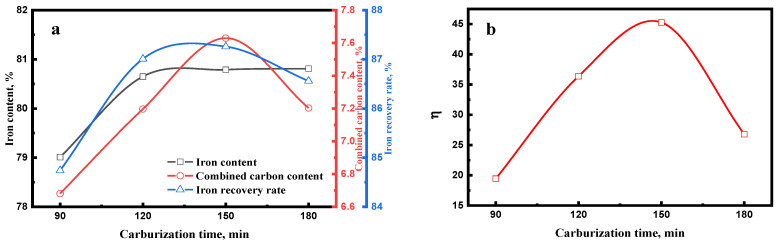
Effect of carburization time on the (**a**) magnetic concentrate and (**b**) carburization index of carburized pellet (carburized at 700 °C min with 20% Na_2_SO_4_).

**Figure 11:**
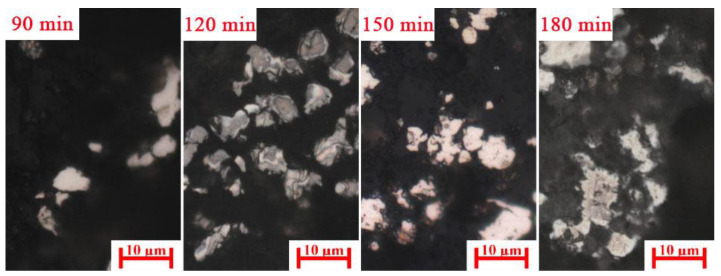
Microstructures of carburized pellet at different carburization times (iron carbide: white).

Figure 10a shows the effect of carburization time on the magnetic concentrate. The iron content, combined carbon content, and iron recovery rate of magnetic concentrate increased with the increase in the carburization time, whereas the combined carbon and iron recovery rate decreased when the carburization time was over 150 min. With the increase in carburization time, the carburization index increased (Figure 10b). A high carburization index shows a high iron carbide content. As show in Figure 11, the iron carbide particle size increased with the increasing carburization time. A high iron carbide content and large iron carbide particle improved the separation efficiency of iron carbide and gangue.

When the carburization time was over 150 min, the iron carbide decomposed, which led to the low carburization index (Figure 10b), which indicated the low iron carbide content. However, the size of iron carbide particles continued to increase. Thus, the combined carbon content and the recovery rate of magnetic concentrate decreased and the iron content of magnetic concentrate slightly increased.

### 3.2. Preparation of Carbon Concentrate

#### Effect of Acetic Acid Concentration

**Figure 12:**
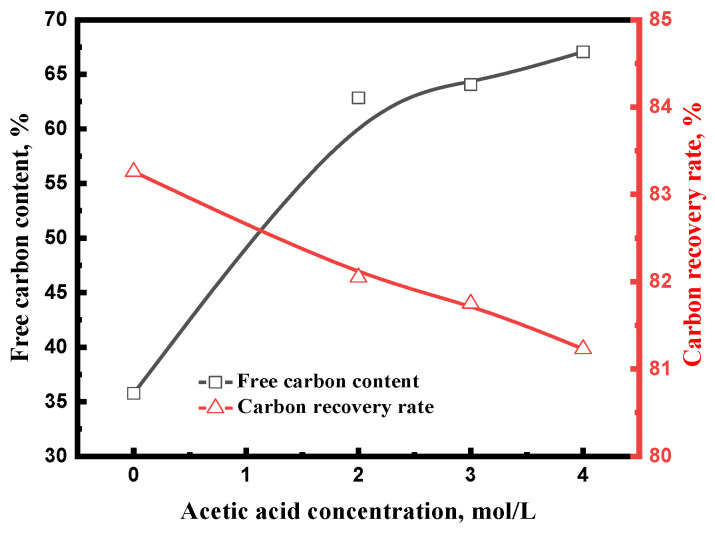
Effect of acetic acid concentration on the acid leaching concentrate (carburized at 700 °C for 150 min with 20% Na_2_SO_4_; leached at 70 °C for 60 min).

Figure 12 shows the influence of acetic acid concentration on the free carbon content and carbon recovery rate of acid leaching concentrate. Without acid leaching, the free carbon content and carbon recovery rate of acid leaching concentrate were 35.77% and 83.26%, respectively. When the tailing was leached in the acetic acid, the free carbon content of acid leaching concentrate significantly increased, whereas the carbon recovery rate of acid leaching concentrate slightly decreased. Compared with the tailing without acid leaching, the free carbon content and carbon recovery rate of acid leaching concentrate reached 67.06% and 81.23%, respectively, when the acetic acid concentration was 4 mol/L.

**Figure 13:**
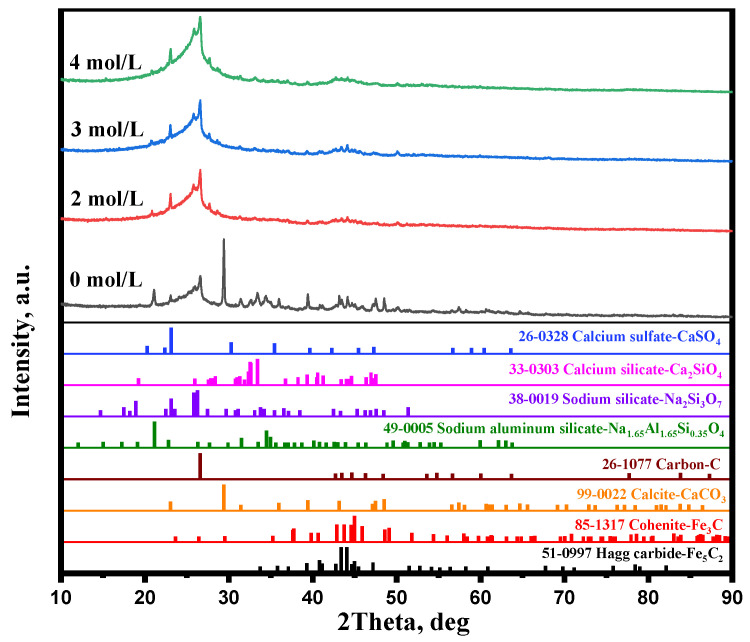
XRD patterns of acid leaching concentrate with different acetic acid concentrations.

It is found from Figure 13 that the gangues, such as CaCO_3_, Ca_2_SiO_4_, CaSO_4_, Na_2_Si_3_O_7_, and Na_1.65_Al_1.65_Si_0.35_O_4_, were produced during the carburization process. In addition to these gangues, a small amount of iron carbide remained in tailing. The XRD results of acid leaching concentrate show that the diffraction peak intensities of CaCO_3_, Na_2_Si_3_O_7_, Na_1.65_Al_1.65_Si_0.35_O_4_, Ca_2_SiO_4_, Fe_3_C, and Fe_5_C_2_ significantly decreased, whereas the diffraction peak intensities of C and CaSO_4_ increased, especially C. It is clearly found that CaCO_3_, Na_2_Si_3_O_7_, Na_1.65_Al_1.65_Si_0.35_O_4_, and Ca_2_SiO_4_ dissolved in the acetic acid, while free carbon and CaSO_4_ did not dissolve in the acetic acid. It is speculated that H^+^ reacts with CaCO_3_, Ca_2_SiO_4_, Na_2_Si_3_O_7_, and Na_1.65_Al_1.65_Si_0.35_O_4_ (Equations (9)–(12)) during the acid leaching process. The different solubility of these gangues and free carbon in acetic acid enhanced the separation efficiency of free carbon and gangue significantly. The carbon phase can be easily enriched by removing Ca, Si, Al, and Na in the form of sodium aluminosilicates, sodium silicate, dicalcium silicate, and calcium carbonate by acid leaching treatment. In addition, the XRD results of acid leaching concentrate show that the diffraction peak intensities of Fe_3_C and Fe_5_C_2_ dramatically decreased, which indicated that Fe_3_C and Fe_5_C_2_ also dissolved in the acetic acid (Equations (13) and (14)). The dissolution of these iron carbides also improved the free carbon content of acid leaching concentrate.

(9) $$CaCO_{3}+2CH_{3}COOH_{\left(aq\right)}\to {\left(CH_{3}COO\right)}_{2}Ca_{\left(aq\right)}+CO_{2}+H_{2}O_{\left(aq\right)}$$

(10) $$Ca_{2}SiO_{4}+4CH_{3}COOH_{\left(aq\right)}\to 2{\left(CH_{3}COO\right)}_{2}Ca_{\left(aq\right)}+H_{2}SiO_{3}{}_{\left(aq\right)}+H_{2}O_{\left(aq\right)}$$

(11) $$xNa_{2}O\cdot yAl_{2}O_{3}\cdot zSiO_{2}{}_{\left(S\right)}+CH_{3}COOH_{\left(aq\right)}\to {\left(CH_{3}COO\right)}_{3}Al_{\left(aq\right)}+CH_{3}COONa_{\left(aq\right)}+H_{2}SiO_{3}{}_{\left(aq\right)}$$

(12) $$Na_{2}Si_{3}O_{7}+2CH_{3}COOH_{\left(aq\right)}+2H_{2}O_{\left(aq\right)}\to 2CH_{3}COONa_{\left(aq\right)}+3H_{2}SiO_{3}{}_{\left(aq\right)}$$

(13) $$Fe_{3}C+6CH_{3}COOH_{\left(aq\right)}\to 3{\left(CH_{3}COO\right)}_{2}Fe_{\left(aq\right)}+C+3H_{2}$$

(14) $$Fe_{5}C_{2}+10CH_{3}COOH_{\left(aq\right)}\to 5{\left(CH_{3}COO\right)}_{2}Fe_{\left(aq\right)}+2C+5H_{2}$$

### 3.3. Product Analyses

**Figure 14:**
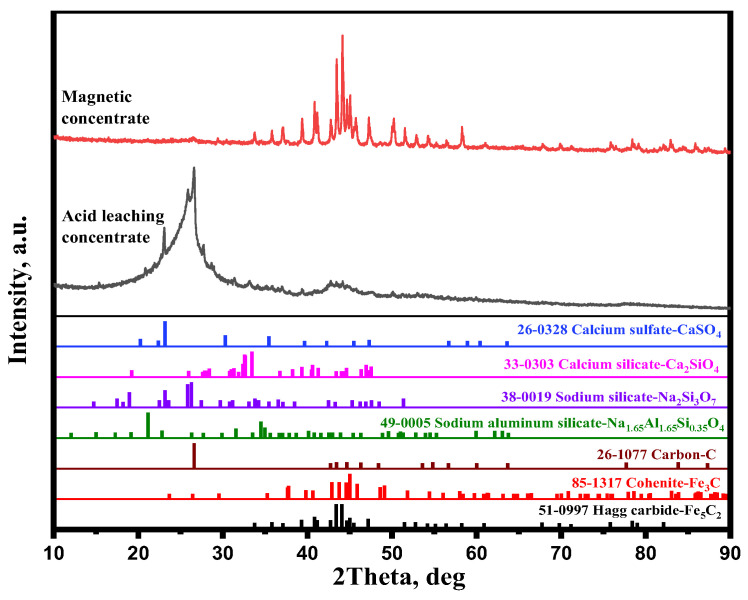
XRD patterns of the magnetic concentrate and acid leaching concentrate.

After carburization at 700 °C for 150 min with 20% Na_2_SO_4_ and leaching at 70 °C for 60 min with 4 mol/L acetic acid, the magnetic concentrate and acid leaching concentrate were ready for further analyses. Figure 14 shows the XRD results corresponding to the magnetic concentrate and acid leaching concentrate. It is apparent that Fe_5_C_2_ was the main mineral phase and Fe_3_C was the minor mineral phase. Aside from Fe_5_C_2_ and Fe_3_C, a small amount of carbon remained in the magnetic concentrate. It can be concluded that the separation degree of iron carbide from gangue was good. The XRD results of the acid leaching concentrate show the diffraction peak intensity of C was strong, whereas those of Ca_2_SiO_4_, CaSO_4_, Na_2_Si_3_O_7_, Na_1.65_Al_1.65_Si_0.35_O_4_, Fe_5_C_2_, and Fe_3_C were weak and the diffraction peak of CaCO_3_ disappeared. It is clear that the main mineral component was C in the acid leaching concentrate, with smaller amounts of Ca_2_SiO_4_, CaSO_4_, Na_2_Si_3_O_7_, Na_1.65_Al_1.65_Si_0.35_O_4_, Fe_5_C_2_, and Fe_3_C also being present.

**Figure 15:**
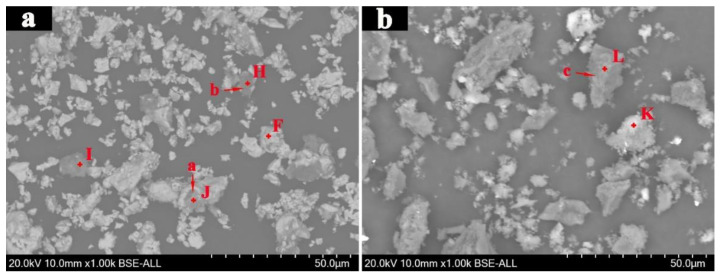
SEM images of the (**a**) magnetic concentrate and (**b**) acid leaching concentrate.

Figure 15 shows the SEM images of the magnetic concentrate and acid leaching concentrate, and the EDS results are listed in Table 2. It is clearly found that the magnetic concentrate was very fine and the particle size was mostly less than 10 μm. Owing the fine grinding, these fine iron carbides and gangue were strongly liberated. However, there was still a small amount of large iron carbide particles (particle a). These iron carbides and gangue cannot be liberated, and they remained interconnected. In addition, fine iron carbide and gangue particles can easily reunite during the magnetic separation process, which led to a smaller number of fine gangue particles (particle b) remaining in the magnetic concentrate. From Table 2 and Figure 14, the gangue particles were fine carbon particles or mixed particles of carbon, Na_2_Si_3_O_7_, and Na_1.65_Al_1.65_Si_0.35_O_4_. Therefore, it can be concluded that the separation efficiency of iron carbide and gangue was good and the separation efficiency of iron carbide and gangue may be further increase if the magnetic separation process continues to be optimized.

From Figure 15b, most gangue particles were also very fine. These fine gangue particles and fine carbon particles aggregated together (spot K). Abundant carbon particles were large (particle c). These large carbon particles were also mixed with some gangue (spot L). As shown in Figure 14 and Table 2, these gangues contained Ca_2_SiO_4_, CaSO_4_, Na_2_Si_3_O_7_, and Na_1.65_Al_1.65_Si_0.35_O_4_. Owing to the weak acidity of acetic acid, residual gangue in acid leaching concentrate was hard to further remove. Acid leaching teat with other acids should be carried out, if the separation efficiency of carbon and gangue is to be further enhanced.

## 4. Conclusions

BF dust was carburized to iron carbide using a CO–H_2_ gas mixture and, subsequently, the iron carbide and carbon concentrates were purified by magnetic separation and acid leaching, respectively. The major conclusions were as follows.

(1) The carburization roasting/magnetic separation/acid leaching process was a highly effective technology to treat BF dust and it suitably recovered Fe and C. An EAF burden assaying 80.79% Fe and 7.63% C with a corresponding iron recovery rate of 87.26% and a carbon concentrate assaying 67.06% C with a corresponding carbon recovery rate of 81.23% were prepared by the carburization of BF dust pellet at 700 °C for 150 min with the addition of 20% Na_2_SO_4_, followed by magnetic separation and acid leaching.
(2) Magnetic separation and acid leaching were useful for purifying the iron carbide and carbon concentrate. Magnetic separation treatment can effectively separate iron carbide from gangue. Acid leaching can usefully treat magnetic separation tailing. Carbon can be separated from gangue using acid leaching treatment.
(3) Na_2_SO_4_ additive in BF dust was a feasible measure to enhance the separation efficiency among iron carbide, carbon, and gangue. The study indicates that Na_2_SO_4_ can enhance the carburization index, enlarge the iron carbide particle size, and improve the embed embedded relationship of iron carbide and gangue, which dramatically improves the separation efficiency of iron carbide and gangue in magnetic separation. In addition, gangue that cannot dissolve in acid translates to gangue that is dissolvable in acid by the sodium modification reactions, so it significantly promotes the separation efficiency of carbon and gangue during the acid leaching process.

This work identified that a high-quality EAF burden could be prepared from BF dust, which can replace scrap during the EAF steelmaking. Compared with the traditional technology, CO_2_ emission can be reduced owing to the CO and H_2_ gas used in our technology. However, the carbon content of carbon concentrate was not high, which may only be used as a steam coal. In future work, the carbon content of carbon concentrate should be significantly improved, which can be used in BF ironmaking or as a coking coal. Moreover, the strength of carburized pellets should be investigated in order to fit the industrial production. Owing to the acid leaching process, acid leaching filtrate may pollute the environment. In order to reduce the risk of environment pollution and recover valuable elements, such as Si and Al, acid leaching filtrate should be further treated.

## Figures and Tables

**Table 1:** The main chemical composition of BF dust, wt.%.

TFe	SiO_2_	Al_2_O_3_	CaO	MgO	MnO	P	S	C	LOI *
32.25	6.03	2.95	4.03	0.87	0.25	0.05	0.15	34.00	37.25

* Loss on ignition.

**Table 2:** EDS results of the magnetic concentrate and acid leaching concentrate, wt.%.

Composition	Fe	C	O	Al	Si	Mg	Ca	K	Na	S
Spot F	84.2	15.8	/	/	/	/	/	/	/	/
Spot H	2.7	89.9	6.3	/	0.2	/	/	0.2	0.3	0.4
Spot I	9.7	38.4	32.8	5.6	7.0	1.9	/	/	4.6	/
Spot J	36.8	31.5	21.6	/	1.8	/	3.3	/	1.2	3.7
Spot K	3.4	46.9	37.8	1.4	9.7	/	0.4	/	/	0.4
Spot L	/	73.2	20.6	2.0	2.7	/	/	0.6	0.5	0.4

## Data Availability

Not applicable.
